# Posterior Reversible Encephalopathy Syndrome as Presenting Form of Very Early Systemic Sclerosis

**DOI:** 10.1155/2015/290378

**Published:** 2015-02-23

**Authors:** María Isabel Pedraza, Julia Barbado, Marina Ruiz, Ángel Luis Guerrero

**Affiliations:** ^1^Neurology Department, Hospital Clínico Universitario, Avenida Ramón y Cajal 3, 47005 Valladolid, Spain; ^2^Internal Medicine Department, Hospital Clínico Universitario, Avenida Ramón y Cajal 3, 47005 Valladolid, Spain

## Abstract

*Introduction*. Posterior Reversible Encephalopathy Syndrome (PRES) is an increasingly recognized clinical and radiological entity with a wide spectrum of symptoms. Its mechanism depends on failure of the blood-brain barrier due to high systemic blood pressure (BP) and loss of integrity of vascular endothelium related with different triggers. *Methods*. We aim to report a case of PRES induced by arterial hypertension and very early systemic sclerosis (SSc) not previously known. *Results*. A 64-year-old female was admitted due to 1-week pulsating headache more prominent on frontal scalp, accompanied by phonophobia, photophobia, and facial flushing. Neurological exam revealed brisk deep tendon reflex. Brain magnetic resonance imaging (MRI) showed subcortical lesions mainly located in posterior regions. BP was monitored and episodic arterial hypertension was detected. In laboratory tests positive anti-topoisomerase I antibodies were detected. BP was controlled with angiotensin-converting-enzyme inhibitors and headache improved. In a new MRI a month later improvement of white matter lesions was observed. Capillaroscopy showed “active pattern,” considered typical of SSc. *Conclusion*. In SSc anti-endothelial cell antibodies impair vascular endothelium and liberation of vasoconstrictors leads to BP increasing and disruption of blood-brain barrier autoregulation mechanisms. PRES can be the first manifestation of very early SSc and this entity should be considered even in absence of skin lesions or Raynaud phenomenon.

## 1. Introduction

Posterior Reversible Encephalopathy Syndrome (PRES) is an increasingly recognized clinical and radiological entity with a wide spectrum of symptoms [1, 2]. Typical neuroimaging consists of leukoencephalopathy in posterior regions [3]. Pathogenesis of this disorder includes failure of the blood-brain barrier due to high systemic blood pressure (BP) as well as loss of integrity of vascular endothelium related with different triggers. We report a case of PRES associating arterial hypertension and a very early systemic sclerosis (SSc) not previously known.

## 2. Case Report

**Figure 1:**
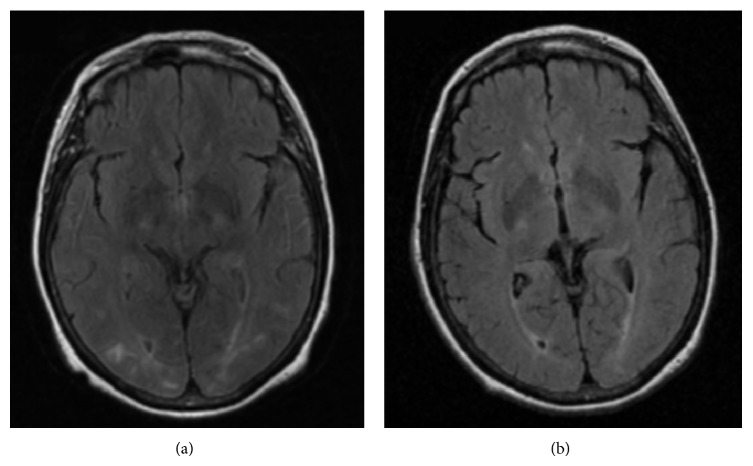
FLAIR MRI. (a) Subcortical lesions mainly located in posterior regions. (b) A month later an improvement of white matter lesions was observed.

**Figure 2:**
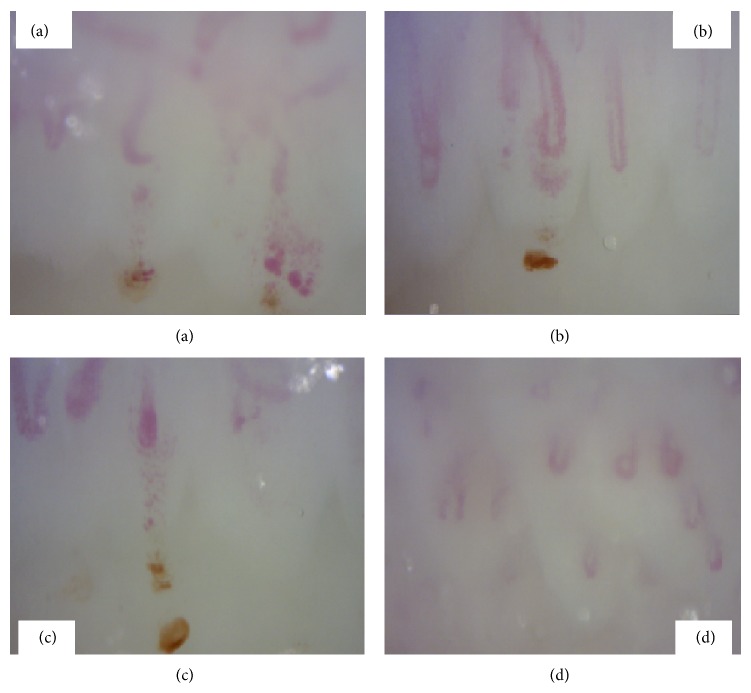
Capillaroscopy. (a) Moderate loss of capillaries. (b) Giant capillaries. (c) Capillary microhemorrhages. (d) Granular flow.

A 64-year-old woman with a history of occasional arterial hypertension with no medical treatment and episodic headache with nauseas and vomiting was admitted due to one-week history of pulsating headache, especially prominent on frontal scalp and accompanied by phonophobia, photophobia, and facial flushing with blood pressure of 175/76 mmHg. Pain worsened with cephalic movement and improved in the darkness. Physical examination on admission including lung, heart, skin, and joint did not show any alteration. No Raynaud phenomenon was described and observed. Neurological examination revealed brisk deep tendon reflex with increased reflexogenic area in four limbs. Fundoscopy was normal, as well as cranial computed tomography (CT). The laboratory examination performed in the emergency department showed normal hemogram with hemoglobin level of 13.5 gr/dL and normal biochemistry with creatinine level of 0.71 mg/dL. Brain magnetic resonance imaging (MRI) revealed subcortical hyperintensities in FLAIR and diffusion-weighted sequences, mainly located in posterior regions (Figure 1(a)). Carotid ultrasound imaging and transcranial duplex showed a generalized increase in flow velocity. BP was monitored and elevated BP episodes were detected. BP was controlled with enalapril and headache improved. Laboratory tests showed the following data: creatinine, 0.8 mg/dL, LDH, 275 U/L, plaque count, 153000, ESR, 10 mm, and CPR, 2.37 mg/L. The immunology test only revealed the presence of antinuclear antibodies 1/80 with positive anti-topoisomerase I (scl-70) antibodies. In a new brain MRI a month later an improvement of white matter lesions in FLAIR and no abnormalities in diffusion-weighted imaging (Figure 1(b)) were observed. Finally capillaroscopy (Figure 2) showed “active pattern” with frequent giant capillaries, moderate loss of capillaries, mild disorganization of the capillary architecture, frequent capillary microhemorrhages, and background edema; all of these findings are considered typical of SSc.

A year and a half after this admission, telangiectasias on hands appeared and our patient began to suffer a trigeminal neuralgia, thus fulfilling EUSTAR criteria.

## 3. Discussion

Etiology of PRES involves a failure of cerebral autoregulation in the setting of severe hypertension along with endothelial injury. Many etiologies have been described in relation to PRES sharing a possible endothelial dysfunction, as autoimmune inflammatory diseases, pregnancy-related conditions, organ transplantation, chronic renal failure, dialysis, neurologic illness as Guillain-Barre syndrome, spinal cord and head injury, and certain drugs, mainly immunosuppressive therapy [4]. This can lead to a breakdown in blood-brain barrier with cerebral edema or cerebral vasospasm with resulting ischemia.

Although headache, acute and subacute confusion, seizures, and visual disturbances are symptoms classically described in PRES, this syndrome may mimic other neurological disorders which should be ruled out [1, 2, 5].

Spectrum of imaging findings in PRES is wide. Cortical and subcortical structures are commonly affected, more frequently in posterior regions with bilateral distribution. Involvement of frontal or temporal lobes, basal ganglia, cerebellum, or brain stem has also been described. On CT imaging, these lesions appear as diffuse hypodense areas and in MRI as high signal intensity on T2 and FLAIR and low signal intensity in T1 sequences. In some cases contrast enhancement and hyperintensities in diffusion-weighted imaging were reported [6]. ADC mapping shows increased ADC values representing vasogenic edema [6]. Carotid ultrasound imaging and transcranial duplex can show a generalized increase in flow velocity because in PRES there is a vascular endothelial injury with focal or diffuse vasoconstriction and focal vasodilatation or a “string-of-beads” appearance in angiography [5].

SSc, also referred to as scleroderma, is a heterogeneous connective tissue disease characterized by skin fibrosis and internal organ involvement. Endothelial injury with vascular dysfunction is an early event in the development of SSc. Specific autoantibodies, such as extractable nuclear antigens (ENA), aid in the diagnosis; one of them, anti-Scl-70, is commonly associated with diffuse cutaneous systemic antibodies, pulmonary fibrosis, and digital ulcers. This antibody was present in our patient together with antinuclear antibodies; finally a capillaroscopy permitted us a direct visualization of digital microvasculature and very early systemic sclerosis was confirmed according to criteria proposed by EUSTAR (EULAR Scleroderma Trial and Research) group [7].

Neurological involvement in SSc is uncommon and includes peripheral neuropathy or trigeminal neuralgia. We have only found two previous reports of PRES in SSc patients [8, 9], one with a patient with limited SSc [10], and a series of two patients in which SSc is cited as etiology with no data regarding its systemic involvement [11]. These cases reported on signs such as severe headache as main symptom and hypertension as precipitant, and our description is the only one in which PRES is the presenting symptom of very early SSc [7].

In conclusion, PRES can be the first manifestation of very early SSc and this entity should be considered even in absence of skin lesions or Raynaud phenomenon.

## References

[1] Feske S. K. (2011). Posterior reversible encephalopathy syndrome: a review. *Seminars in Neurology*.

[2] Yasuhara T., Tokunaga K., Hishikawa T. (2011). Posterior reversible encephalopathy syndrome. *Journal of Clinical Neuroscience*.

[3] Hugonnet E., Da Ines D., Boby H. (2013). Posterior reversible encephalopathy syndrome (PRES): features on CT and MR imaging. *Diagnostic and Interventional Imaging*.

[4] Dhillon A., Velazquez C., Siva C. (2012). Rheumatologic diseases and posterior reversible encephalopathy syndrome: two case reports and review of the literature. *Rheumatology International*.

[5] Stevens C. J., Heran M. K. S. (2012). The many faces of posterior reversible encephalopathy syndrome. *British Journal of Radiology*.

[6] Kastrup O., Schlamann M., Moenninghoff C., Forsting M., Goericke S. (2014). Posterior reversible encephalopathy syndrome: the spectrum of MR imaging patterns. *Clinical Neuroradiology*.

[7] Hudson M., Fritzler M. J. (2014). Diagnostic criteria of systemic sclerosis. *Journal of Autoimmunity*.

[8] Hounoki H., Shinoda K., Taki H., Ogawa R., Sugiyama E., Tobe K. (2011). Reversible posterior leukoencephalopathy syndrome in a patient with systemic sclerosis. *Journal of Clinical Rheumatology*.

[9] Del castillo-piñol N., Robustillo-Villarino M., Narváez J. A., Narvàez García F. J. (2010). Reversible posterior leukoencephalopathy in diffuse scleroderma. *Clinical and Experimental Rheumatology*.

[10] Poon W. L., Mok C. C. (2005). Reversible posterior leucoencephalopathy in scleroderma. *Annals of the Rheumatic Diseases*.

[11] Fugate J. E., Claassen D. O., Cloft H. J., Kallmes D. F., Kozak O. S., Rabinstein A. A. (2010). Posterior reversible encephalopathy syndrome: associated clinical and radiologic findings. *Mayo Clinic Proceedings*.

